# Clinicopathologic Characteristics of Somatic *TP53* Mutations in Philadelphia Chromosome–Negative Myeloproliferative Neoplasms: A Systematic Review

**DOI:** 10.1002/jha2.70385

**Published:** 2026-09-19

**Authors:** Mahmood Aldapt, Rasha Kaddoura, Ahmed O. Saleh, Mostafa Najim, Ahmad Salem, Abdulrahman F. Al‐Mashdali, Bassam Muthanna, Ruba Adel Aweer, Marrita Rabadi, Shehab F. Mohamed

**Affiliations:** ^1^ Division of Hematology and Medical Oncology Mayo Clinic Jacksonville Florida USA; ^2^ Department of Pharmacy Heart Hospital Hamad Medical Corporation Doha Qatar; ^3^ Department of Internal Medicine Unity Hospital/Rochester Regional Health Rochester New York USA; ^4^ Department of Internal Medicine Penn State – M.S. Hershey Medical Center Hershey Pennsylvania USA; ^5^ Division of Hospital Medicine The Ohio State University Wexner Medical Center Columbus Ohio USA; ^6^ Department of Hematology National Center for Cancer Care and Research Hamad Medical Corporation Doha Qatar; ^7^ Department of Internal Medicine University of Missouri Columbia Missouri USA; ^8^ College of Medicine Qatar University Doha Qatar

**Keywords:** accelerated/blast phase, chronic phase, essential thrombocythemia, myelofibrosis, myeloproliferative neoplasm, Philadelphia chromosome–negative, polycythemia vera, TP53‐mutation

## Abstract

**Background:**

*TP53* mutations (*TP53*m) are uncommon in chronic‐phase Ph–negative myeloproliferative neoplasms but become enriched in myelofibrosis (MF) and accelerated/blast phase myeloproliferative (AP/BP‐MPN). Their prevalence, allelic distribution, and phase‐stratified outcomes have not been systematically synthesized.

**Methods:**

A PRISMA‐guided systematic review of EMBASE and PubMed identified cohort studies of adult Ph–negative MPN with molecularly confirmed *TP53*m. Pooled proportions with 95% CI were generated using random‐effects models; survival outcomes were summarized descriptively.

**Results:**

Eleven retrospective cohort studies (*N* =  603) met inclusion criteria. *TP53*m were uncommon overall (3%, 95% CI: 1–13; *I* ^2^ =  97%) but substantially enriched in MF or AP/BP‐MPN cohorts (10%, 95% CI: 4–24; *I* ^2^ =  89%). Among *TP53*m patients, disease distribution included AP/BP‐MPN (33%, 95% CI: 26–41), MF (31%, 95% CI: 15–54), ET (16%, 95% CI: 5–41), and PV (11%, 95% CI: 4–27). Single‐ and multi‐hit *TP53* were present in comparable proportions (52% vs. 48%), with a pooled mean VAF of 37.48% (95% CI: 30.73–44.24; *I* ^2^ =  97%). Unfavorable karyotype was identified in 42%. Median OS was: 37.4–72 months in PV, 44.4–54.6 months in ET, 11.6–24.7 months in MF, and 4.5–6 months in AP/BP‐MPN. In CP‐MPN, multi‐hit *TP53* conferred worse OS (9.5–18.5 months) versus single‐hit disease (38 months to not reached); this distinction was absent in AP/BP‐MPN. Leukemic transformation occurred in 35% (95% CI: 16–60; *I* ^2^ =  93%) across two reporting cohorts, and allo‐HCT was utilized in only 16% (95% CI: 13–19; *I* ^2^ =  12%).

**Conclusions:**

*TP53*m Ph–negative MPN are enriched in advanced‐phase disease, carry comparable proportions of single‐hit and multi‐hit allelic configurations with high clonal burden, and demonstrate a clinically important survival gradient. Allelic status is a key prognostic determinant in CP‐MPN but loses significance in AP/BP‐MPN where outcomes are uniformly dismal. Standardized molecular reporting and prospective clinical trials are needed.

**Trial Registration:**

The authors have confirmed clinical trial registration is not needed for this submission.

## 1 Introduction

Philadelphia (Ph) chromosome–negative myeloproliferative neoplasms (MPNs)—polycythemia vera (PV), essential thrombocythemia (ET), and primary or secondary myelofibrosis (MF)—are clonal stem‐cell disorders with highly variable clinical course, ranging from indolent disease to rapid progression to accelerated phase (AP) and blast phase (BP). This biological heterogeneity is only partly explained by driver mutations in JAK2, CALR, and MPL, and is further shaped by additional somatic alterations that define “high molecular risk” disease and influence both survival and risk of leukemic transformation [1, 2, 3]. Among these, *TP53* alterations are of particular interest because of their central role in maintaining genomic stability and their well‐established association with treatment resistance and dismal outcomes in myelodysplasia (MDS) and acute myeloid leukemia (AML). The International Consensus Classification (ICC) and World Health Organization (WHO) fifth edition now recognize *TP53*‐mutated myeloid neoplasms as distinct entities, underscoring the uniformly poor prognosis of these patients [4, 5, 6].

In MPN, *TP53*‐mutations appear to be relatively uncommon in the chronic phase (CP) but become enriched during disease evolution. Large sequencing series and registry studies suggest that *TP53* mutations are present in roughly 2%–8% of CP‐MPN, but in up to ∼30% of AP/BP‐MPN cases [7, 8, 9, 19]. Farnoud et al. first outlined the “landscape” of *TP53* mutations across MPN subtypes, reporting a broad spectrum of missense and truncating variants, often with high variant allele frequency (VAF) and frequent association with complex karyotype in advanced disease [19]. Ultra‐deep sequencing has also revealed that low‐burden *TP53* clones (VAF as low as 0.2%) can be detected in a substantial proportion of patients receiving cytoreductive therapy and may gradually expand over time, suggesting a multistep trajectory from small *TP53*‐mutated subclones to overt leukemic transformation [8].

Recent work has refined the concept of allelic status in *TP53*‐mutated MPN. Tefferi and coworkers, in a large multi‐center cohort, showed that the clinical impact of *TP53* mutations is strongly context dependent: in MF, multi‐hit *TP53* was associated with a median overall survival (OS) of around 10 months, compared with approximately 35 months for single‐hit *TP53*, whereas in AP/BP‐MPN any *TP53* mutation conferred extremely poor outcomes regardless of allelic status [9]. A complementary series by Badar et al. highlighted marked heterogeneity, with low‐VAF, single‐hit *TP53* clones sometimes remaining stable for years in CP disease, while multi‐hit *TP53* or acquisition of additional high‐risk mutations (e.g., RUNX1, SRSF2) heralded rapid progression and death [7]. Fathima et al. further demonstrated that multi‐hit *TP53* in MPN and secondary AML carry uniformly short survival (median: 4–11 months) despite intensive therapy, supporting their inclusion within the broader category of “myeloid neoplasms with mutated *TP53*” [10].

The adverse impact of *TP53‐*mutation also extends to the transplant setting. In a large international series of patients with MF undergoing allogeneic hematopoietic stem‐cell transplantation (allo‐HCT), *TP53*‐mutated cases, particularly those with multi‐hit status, had significantly higher rates of relapse, earlier leukemic transformation and markedly inferior long‐term survival compared with *TP53*‐wild‐type or single‐hit counterparts [11].

Despite these advances, many uncertainties remain. Existing data are dispersed across heterogeneous cohorts with differing inclusion criteria. Moreover, there has been no comprehensive quantitative synthesis of the prevalence of *TP53*‐mutations across MPN subtypes, nor of their pooled impact on survival, leukemic transformation and allo‐HCT utilization.

We therefore designed a systematic review and pooled analysis to characterize the clinicopathologic profile and outcomes of *TP53*‐mutated Ph–negative MPN. Our aims were: to define the frequency of *TP53* mutations across PV, ET, MF, and AP/BP‐MPN; describe their allelic status and VAF level; and summarize associated clinical outcomes including OS, leukemic transformation, and allo‐HCT utilization. A robust synthesis of the available evidence can help refine molecular risk stratification, and guide future clinical trials for patients with *TP53*‐mutated MPN.

## 2 Methods

### 2.1 Literature Search Strategy

This systematic review was conducted in accordance with the PRISMA 2020 guidelines. A comprehensive search of EMBASE (1974–March 1, 2026) and PubMed (inception to March 1, 2026) was performed using controlled vocabulary and free‐text terms related to Ph–negative MPN and *TP53* mutations. The EMBASE strategy incorporated terms for Ph chromosome negativity, *TP53* (protein p53), and all recognized Ph–negative MPN subtypes, including PV, ET, primary MF, chronic neutrophilic leukemia, eosinophilic leukemias, and related entities. A complementary PubMed search used the terms “*TP53* mutation” and “Philadelphia chromosome negative.” Searches were limited to English‐language publications. Records from both databases were combined, and duplicates were removed prior to screening.

### 2.2 Study Selection

After removal of duplicate records, unique articles were screened by title and abstract. Studies were excluded if they were unrelated to the study question, lacked molecular confirmation of *TP53*‐mutations, did not include Ph chromosome–negative MPN, or did not report informative clinical data. Then full‐text articles were assessed for eligibility based on the inclusion and exclusion criteria below. Study selection was performed independently by three reviewers, with discrepancies resolved by consensus.

### 2.3 Eligibility Criteria

Eligible studies were required to include adult patients (age ≥ 18 years) with Ph chromosome–negative MPN harboring a confirmed somatic *TP53*‐mutation detected by next‐generation sequencing (NGS). Only studies recruiting at least six patients were included. Studies were required to report clinical characteristics and/or patient outcomes to be eligible for inclusion. Conference abstracts were not excluded if sufficient cohort‐level data were available. Disease classification followed WHO 2016 and/or WHO 2022 criteria as reported in the original studies. Any discrepancies during study selection were resolved by consensus among the reviewing team.

### 2.4 Data Extraction

Data were independently extracted by two reviewers using a standardized collection framework. Variables collected included patient demographics (age, sex), MPN subtype, disease phase (CP, AP, BP), DIPSS+ risk category (for MF patients), *TP53*‐mutation VAF, cytogenetic risk profile, co‐mutational profile when reported, treatment exposure (including cytoreductive therapy and allo‐HCT), and clinical outcomes. *TP53*‐mutation were identified by NGS in all included studies. Both single‐hit and multi‐hit (per ICC 2022 or WHO definition per the original studies) *TP53* were included. The outcomes of interest included OS, allo‐HCT utilization, and leukemic transformation rate.

### 2.5 Risk of Bias and Missing Data

Risk of bias was assessed using the Newcastle–Ottawa Scale (NOS) for cohort studies, which evaluates three domains: selection (4 items), comparability (2 items), and outcome (3 items), with a maximum score of 9 stars. Studies not providing sufficient methodological detail like letters to the editor and conference abstracts, were not amenable to formal NOS scoring. Variables that were not available in individual studies were reported as such.

### 2.6 Statistical Analysis

Given the retrospective design of all included studies and heterogeneity in study populations, outcome definitions, and reporting practices, a formal comparative meta‐analysis was not feasible. Accordingly, a systematic review with pooled descriptive analysis was performed, summarizing aggregated study‐level data without comparative modeling or effect‐size estimation for the impact of *TP53‐*mutation on the clinical outcomes. Pooled proportions with 95% confidence intervals (CIs) and heterogeneity statistics (*I* ^2^) were generated for continuous and categorical descriptive variables using a random‐effects model. OS was summarized descriptively across studies given the absence of a common comparator group.

## 3 Results

### 3.1 Search Results and Study Characteristics

**FIGURE 1:**
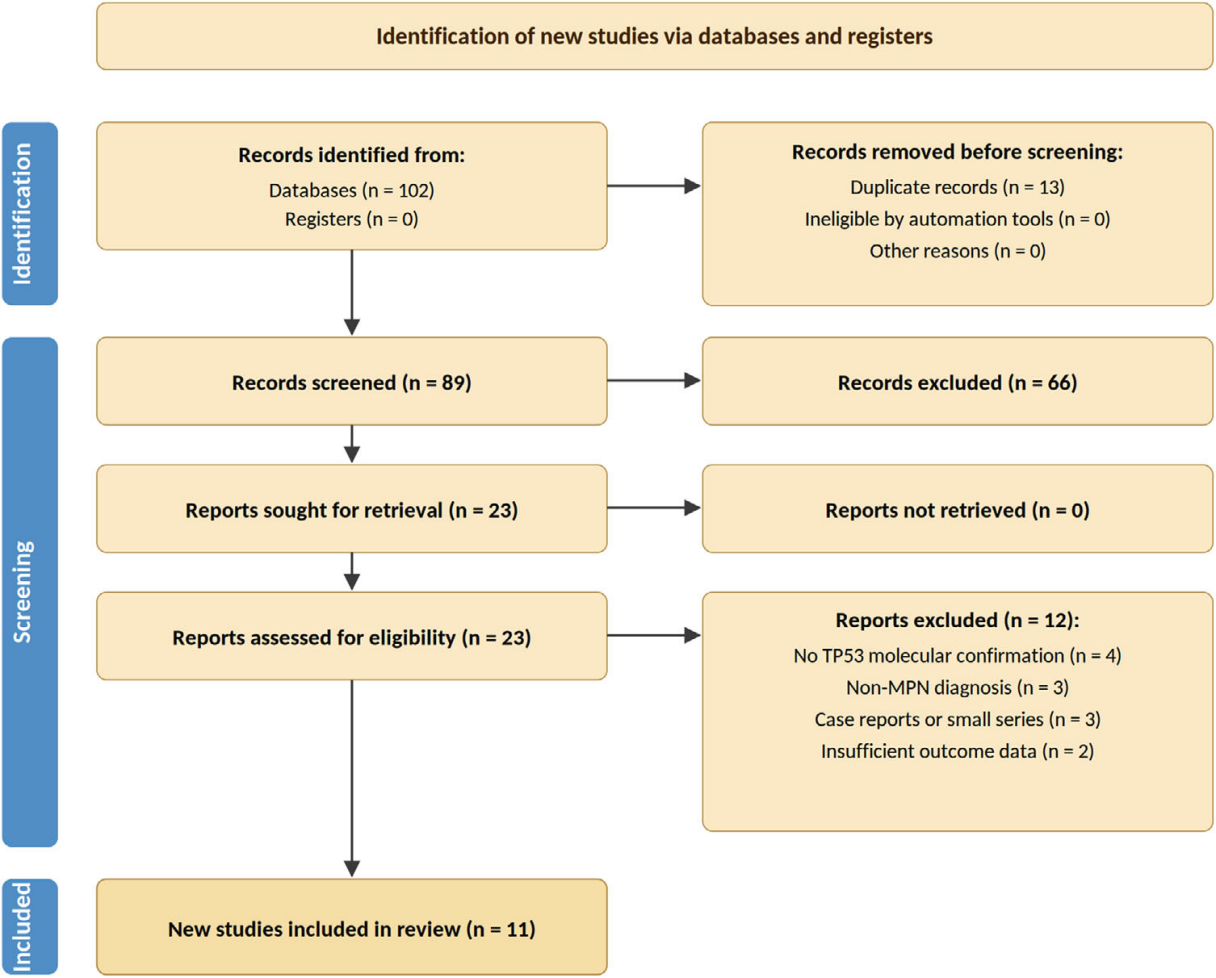
PRISMA 2020 flow diagram of study selection.

The literature search identified 102 records from electronic databases (EMBASE, *n*  =  99; PubMed, *n* = 3). After removal of 13 duplicate records, 89 unique articles were screened by title and abstract, of which 66 were excluded. Twenty‐three full‐text articles were retrieved and assessed for eligibility. Of these, 12 were excluded: reasons included absence of *TP53* molecular confirmation (*n* = 4), non‐MPN diagnosis (*n* = 3), case reports or small series (*n* = 3), and insufficient outcome data (*n* = 2). Eleven retrospective cohort studies met all inclusion criteria and were included in the final pooled descriptive analysis (Figure 1 and Table 1).

**TABLE 1:** Characteristics of included studies of TP53‐mutated Philadelphia chromosome–negative myeloproliferative neoplasms.

Study	Country	Design	Total cohort, *N*	TP53‐mutated, *n* (%)	MPN subtype distribution, *n*	Age, years (media*n*, range)	Male, *n* (%)	Median follow‐up, months	TP53 multi‐hit, *n* (%)	Allogeneic HSCT, *n* (%)
[7]	England	Retrospective	158	158 (100.0)	ET 24; PV 15; PMF 74; AP/BP‐MPN 45	62 (24–86)	NR	NR	65 (41)	23 (22)
[10]	USA	Retrospective	142	142 (100.0)	ET 27; PV 15; PMF 18; AP/BP‐MPN 42	71 (19–94)	79 (56)	11.6	142 (100)	19 (13)
[11]	Multinational	Retrospective	349	49 (14.0)	PMF 245	58 (22–75)	224 (65)*	NR	30 (9)	345 (100)*
[23]	UK	Retrospective	2035	40 (2.0)	ET 1321; PV 356; PMF 309	NR	NR	NR	NR	NR
[13]	Korea	Retrospective	229	8 (3.5)	PMF 122; ET‐MF 46; PV‐MF 21	56.8 (mean)	119 (52)*	32.4	NR	48 (21)*
[14]	USA	Retrospective	1294	8 (0.6)	PMF 456; AP/BP‐MPN 37	NR	NR	NR	NR	NR
[15]	Canada	Retrospective	122	21 (17.2)	PMF 34; ET‐MF 38; PV‐MF 40; AP/BP‐MPN 122	69 (36–86)	72 (59)*	NR	NR	19 (16)*
[16]	USA, Canada	Retrospective, multicenter	1540	111 (7.2)	ET 597; PV 497; PMF 386; AP/BP‐MPN 5; pre‐MF 60	62 (22–93)	59 (53)	100.8	37 (33)	15 (14)
[17]	Italy	Retrospective	92	NR	ET 29; PV 39; PMF 24	70.8 (46.3–86)	NR	NR	NR	NR
[18]	South Korea	Longitudinal	19	3 (15.8)	NR	NR	NR	NR	NR	NR
[9]	USA, Italy, Australia	Retrospective, multicenter	114	114 (100.0)	ET 3; PV 3; PMF 61; AP/BP‐MPN 47	69 (26–88)	64 (54)	9.6	65 (57)	17 (15)

*Note*: Percentages for TP53‐mutated prevalence are calculated using the total cohort as denominator. Percentages for sex, multi‐hit, and HSCT are calculated using the TP53‐mutated subgroup as denominator unless otherwise indicated. Values marked with an asterisk (*) denote that the reported percentage in the source publication used the entire study cohort as the denominator rather than the TP53‐mutated subgroup. All included studies are retrospective cohort studies. MPN subtype distribution reflects the diagnosis at first MPN diagnosis when reported.

Abbreviations: AP, accelerated phase; BP, blast phase; ET, essential thrombocythemia; ET‐MF, post‐ET myelofibrosis; HSCT, hematopoietic stem cell transplantation; MPN, myeloproliferative neoplasm; NR, not reported; PMF, primary myelofibrosis; pre‐MF, prefibrotic primary myelofibrosis; PV, polycythemia vera; PV‐MF, post‐PV myelofibrosis; TP53, tumor protein p53.

### 3.2 Patient Demographics

Across the 11 included studies, the pooled mean age was 61.21 years (95% CI: 57.74–64.68; *I* ^2^ = 96%). Male sex predominated at 57% (95% CI: 53–61; *I* ^2^ = 52%), with females comprising 43% (95% CI: 38–47; *I* ^2^ = 60%) (Table 2 and Figures S1–S3).

**TABLE 2:** Pooled clinicopathologic characteristics of patients with *TP53*‐mutated Philadelphia chromosome–negative MPN.

Variable	Studies (*n*)	Pooled Estimate (95% CI; *I* ^2^)
Mean age (years)	8	61.21 (95% CI: 57.74–64.68) years; *I* ^2^ = 96%
Male sex	6	57% (95% CI: 53; 61, *I* ^2^ = 52%)
Female sex	6	43% (95% CI: 38–47, *I* ^2^ = 60%)
Overall *TP53‐*mutation prevalence in MPN	4	3% (95% CI: 1–13, *I* ^2^ = 97%)
*TP53‐*mutation prevalence in MF or AP/BP‐MPN cohorts	3	10% (4–24; *I* ^2^ = 89%)
AP/BP‐MPN in *TP53* population	3	33% (26–41; *I* ^2^ = 64%)
Primary myelofibrosis in *TP53* population	4	31% (95% CI: 15–54, *I* ^2^ = 95%)
Essential thrombocythemia in *TP53* population	4	16% (95% CI: 5–41, *I* ^2^ = 94%)
Polycythemia vera in *TP53* population	4	11% (95% CI: 4–27, *I* ^2^ = 92%)
MF DIPSS+ High risk	2	36% (4–89; *I* ^2^ = 99%)
MF DIPSS+ intermediate‐2	2	26% (9–57; *I* ^2^ = 94%)
MF DIPSS+ intermediate‐1	2	23% (13–37; *I* ^2^ = 91%)
Multi‐hit *TP53*	4	48% (95% CI: 35–60, *I* ^2^ = 84%)
Single‐hit *TP53*	4	52% (95% CI: 39–65, *I* ^2^ = 85%)
*TP53*‐mutation VAF (mean)	5	37.48% (30.73–44.24; *I* ^2^ = 97%)
Cytogenetic risk—unfavorable	5	42% (11–82; *I* ^2^ = 98%)
Cytogenetic risk—favorable	3	65% (38–85; *I* ^2^ = 97%)
Cytogenetic risk—very high risk	2	6% (4–8; *I* ^2^ = 0%)
Allo‐HCT utilization	6	16% (95% CI: 13–19, *I* ^2^ = 12%)
Median OS in *TP53*‐mutated MF	4	Range: 11.6–24.7 months
Median OS in *TP53*‐mutated PV	2	Range: 37.4–72 months
Median OS in *TP53*‐mutated ET	2	Range: 44.4–54.6 months
Median OS in *TP53*‐mutated AP‐MPN	2	Range: 4.5–5.6 months
Median OS in *TP53*‐mutated BP‐MPN	2	Range: 4.6–6.0 months
Median OS in multi‐hit *TP53* CP‐MPN	3	Range: 9.5–18.5 months
Median OS in single‐hit *TP53* CP‐MPN	3	Range: 38–NR months
Median OS in multi‐hit *TP53* AP/BP‐MPN	2	Range: 5.27–6 months
Median OS in single‐hit *TP53* AP/BP‐MPN	2	Range: 3.5–5.6 months
Leukemic transformation	2	35% (95% CI: 16–60, *I* ^2^ = 93%)

Abbreviations: Allo‐HCT, allogeneic hematopoietic cell transplantation; AP, accelerated phase; BP, blast phase; CI, confidence interval; CP, chronic phase; DIPSS+, Dynamic International Prognostic Scoring System plus; ET, essential thrombocythemia;MF, myelofibrosis; mOS, median overall survival; MPN, myeloproliferative neoplasm; PMF, primary myelofibrosis; PV, polycythemia vera; VAF, variant allele frequency.

### 3.3 *TP53*‐Mutation Prevalence

In overall Ph–negative MPN patients, *TP53*‐mutations were uncommon, with a pooled prevalence of 3% (95% CI: 1%–13%; *I* ^2^ = 97%; Figure S4). However, when restricted to patients with MF or AP/BP‐MPN, the mutation frequency rose substantially to 10% (95% CI: 4%–24%; *I* ^2^ = 89%; Figure S5).

### 3.4 Disease Phase Distribution Among *TP53*‐Mutated Patients

Among patients with confirmed *TP53*‐mutated, AP/BP‐MPN was the most common disease context, accounting for 33% (95% CI: 26%–41%; *I* ^2^ = 64%; Figure S6), followed by MF at 31% of cases (95% CI: 15%–54%; *I* ^2^ = 95%; Figure S7). ET accounted for 16% (95% CI: 5%–41%; *I* ^2^ = 94%; Figure S8) and PV for 11% (95% CI: 4%–27%; *I* ^2^ = 92%; Figure S9).

### 3.5 DIPSS+ Risk Stratification in *TP53*‐Mutated MF

Among *TP53*‐mutated MF patients with available DIPSS+ data, high‐risk disease predominated at 36% (95% CI: 4%–89%; *I* ^2^ = 99%; Figure S10), followed by intermediate‐2 at 26% (95% CI: 9%–57%; *I* ^2^ = 94%; Figure S11) and intermediate‐1 at 23% (95% CI: 13%–37%; *I* ^2^ = 91%; Figure S12).

### 3.6 *TP53* Allelic Status, VAF, and Cytogenetic Risk

Multi‐hit *TP53* accounted for 48% (95% CI: 35%–60%; *I* ^2^ = 84%; Figure S13) and single‐hit for 52% (95% CI: 39%–65%; *I* ^2^ = 85%; Figure S14). The pooled mean *TP53* VAF was 37.48% (95% CI: 30.73%–44.24%; *I* ^2^ = 97%; Figure S15). Regarding cytogenetic risk, it was inconsistently reported across the studies, very high risk was reported in only 6% (95% CI: 4%–8%; *I* ^2^ = 0%; Figure S16) of patients with *TP53*‐mutation, whereas, unfavorable karyotype was present in 42% (95% CI: 11%–82%; *I* ^2^ = 98%; Figure S17) and favorable karyotype was present in 65% (95% CI: 38%–85%; *I* ^2^ = 97%; Figure S18). Cytogenetic risk was not uniformly reported across studies; each pooled estimate reflects a different subset of contributing studies.

### 3.7 Clinical Outcomes

*Allo‐HCT Utilization*: Allo‐HCT was employed in a pooled 16% of *TP53*‐mutated patients (95% CI: 13%–19%; *I* ^2^ = 12%; Figure S19).

OS *by Allelic Status*: Three studies reported median OS stratified by *TP53* allelic status. In CP‐MPN: Tefferi et al. reported median OS of 10.0 months in multi‐hit *TP53* compared to 35 months in single‐hit *TP53*. Badar et al. reported 18.5 months in multi‐hit versus not reached median OS in single‐hit, while Rolles et al. reported median OS of 9.6 versus 38.4 months, respectively. In AP/BP‐MPN, Badar et al. reported median OS of 5.27 months in multi‐hit versus 5.6 months in single‐hit, while Tefferi et al. reported median OS of 6 months in multi‐hit versus 3.5 months in single hit.

OS *by Disease Subtype*: Tefferi et al. reported median OS of 18.0 months in MF, NR in PV/ET, 6.0 months in BP‐MPN, and 4.5 months in AP‐MPN. Fathima et al. reported median OS of 11.6 months in MF, 4.6 months in BP‐MPN, and 5.6 months in AP‐MPN. Badar et al. reported median OS of 37.4 months in PV, 54.6 months in ET, 24.7 months in MF, and 5.4 months in AP/BP‐MPN combined. Finally Rolles et al. reported median OS of 13.2 months in MF, 72 months in PV, and 44.4 months in ET.

*Leukemic Transformation*: AML transformation data within *TP53*‐mutated patients was reported in two studies. Pooled analysis showed leukemic transformation rate of 35% (95% CI: 16–60, *I* ^2^ = 93%; Figure S20).

### 3.8 Risk of Bias

NOS scoring was applicable to 7 of the 11 included studies; Badar et al. and Marcellino et al. were published as letters to the editor without sufficient methodological detail, and Roncoroni et al. and Son et al. were conference abstracts, precluding formal assessment. Among the seven assessable studies, NOS scores ranged from 7 to 8, and all were classified as low risk of bias. All seven studies fulfilled criteria for ascertainment of exposure, demonstration that the outcome of interest was not present at study initiation, comparability on the basis of age and additional controlled factors, outcome assessment, and adequate follow‐up duration for outcomes to occur. Adequacy of follow‐up of cohorts was not met in any of the seven assessed studies. Overall, the formally assessable studies demonstrated acceptable methodological quality (Table S1).

## 4 Discussion

This systematic review represents, to our knowledge, the first comprehensive synthesis of the clinicopathologic characteristics and outcomes of somatic *TP53*‐mutations across the full spectrum of Ph–negative MPN, addresses three major points: to define the frequency of *TP53* mutations across MPN subtypes and disease phases; to characterize their allelic status and VAF level; and to illustrate their associated clinical outcomes including OS, leukemic transformation, and allo‐HCT utilization. The findings establish that *TP53*‐mutations in Ph–negative MPN are not uniformly distributed or predict the same outcome; rather, their clinical significance is shaped by disease phase, and allelic status, a paradigm that has direct implications for risk stratification, therapeutic decision‐making, and trial design.

### 4.1 Prevalence and Disease‐Phase Enrichment

The low overall prevalence of *TP53*‐mutations in overall MPN (3%, 95% CI: 1%–13%) is consistent with established single‐institution estimates. Tefferi et al. reported *TP53‐*mutations incidence of 2.6% in PV and 1.7% in ET in a consecutively screened Mayo Clinic cohort 9]. The uniformly high *I* ^2^ values across prevalence estimates reflect study‐level heterogeneity in cohort composition.

### 4.2 DIPSS+ Risk Distribution and Clinical Implications

The strong enrichment of intermediate‐2 and high‐risk DIPSS+ categories among *TP53*‐mutated MF patients suggests that *TP53*‐mutations are more likely to be detected in patients who are already at higher risk, and conversely, their acquisition likely accelerates based on this high risk. The extremely high heterogeneity (*I* ^2^ = 91%–99%) and wide CIs reflect the limited number of contributing studies (*n* = 2) and should be regarded as exploratory. Current prognostic calculators do not account for *TP53* allelic status or VAF, and the data presented here, though limited by small contributing study numbers, suggest that the molecular risk conferred by *TP53* likely operates independently of and additively to DIPSS+ score. More studies examining whether *TP53* status modifies DIPSS+‐defined risk categories are needed to answer this question.

### 4.3 Allelic Status, VAF, and Cytogenetic Risk

The 48% prevalence of multi‐hit *TP53*‐mutations in this pooled MPN analysis is notably lower than that reported in large MDS cohorts, where biallelic or multi‐hit configurations account for approximately two‐thirds of *TP53*‐mutated cases [12]. The pooled mean VAF of 37.48%; well above the 20%–23% threshold identified as prognostically impactful [12], further underscores the clonal dominance of *TP53*‐mutated cells in the included cohorts and is consistent with the advanced disease phases that predominated in contributing studies. Regarding cytogenetic risk, estimates were inconsistently reported across studies, each figure reflects a different subset of contributing studies and should be interpreted independently rather than as components of a single additive distribution. The pooled proportions do not sum to 100% because each estimate was derived from a different subset of studies that reported the corresponding subgroup.

### 4.4 Survival Outcomes

The phase‐dependent survival pattern emerging from this synthesis, with median OS of 37.4–72 months in PV, 44.4–54.6 months in ET, 11.6–24.7 months in MF, and 4.5–6 months in AP/BP‐MPN, directly answers our aim to characterize outcomes across the MPN disease spectrum and reveals a clinically important gradient. The median OS of 11.6–24.7 months in MF overlaps with the 8.7 month median OS reported for multi‐hit *TP53*‐mutated MDS [12], suggesting that *TP53*‐mutated MF and higher‐risk MDS occupy a comparable prognostic outcomes. In contrast, *TP53*‐wild‐type higher‐risk MF in the ruxolitinib era carries a median OS exceeding 3 years [20], confirming that *TP53*‐mutations define a biologically and prognostically distinct MF subset that is not captured by DIPSS+ alone. At the most severe end, the median OS of 4.5–6 months in AP/BP‐MPN is comparable to that of *TP53*‐mutated AML, where median OS with current therapies ranges from 5 to 10 months regardless of treatment intensity [21].

The observation that the survival advantage of single‐hit over multi‐hit *TP53* essentially disappears in AP/BP‐MPN (5.27 vs. 5.6 months, Badar et al.) is particularly informative. We hypothesize that this finding reflects the emergence of additional high‐risk molecular co‐events that override the prognostic influence of *TP53* allelic status once blast transformation has occurred, a pattern analogous to what has been observed in *TP53*‐mutated AML where multi‐hit status has a smaller relative impact than in MDS [22]. In chronic‐phase disease, where the genomic landscape is less complex, allelic state retains its value.

### 4.5 Leukemic Transformation

The pooled 35% leukemic transformation rate (95% CI: 16–60; *I* ^2^ = 93%) observed across two reporting cohorts underscores the aggressive natural history of *TP53*‐mutated MPN and is concordant with transformation rates reported in *TP53*‐mutated MDS, where AML evolution is a defining feature of the disease course [21]. The critical gap is that 9 of the 11 included studies did not report transformation rates in the *TP53*‐mutated subset at all, making it impossible to estimate the true transformation risk. Future prospective registries in *TP53*‐mutated MPN must mandate transformation rate as an endpoint.

### 4.6 Allo‐HCT Utilization

The consistent 16% allo‐HCT utilization rate across contributing centers (*I* ^2^ = 12%) signals that this under‐utilization is a systemic pattern rather than a center‐specific phenomenon, and likely reflects clinical dilemma about transplant benefit in *TP53*‐mutated MPN rather than, age, performance status, and comorbidities alone. Gagelmann et al. demonstrated that *TP53*‐mutated MF patients have significantly higher relapse rates and inferior posttransplant survival compared with wild‐type counterparts [11], yet also showed that a subset achieves durable remission. Novel conditioning strategies incorporating hypomethylating agents, and posttransplant maintenance approaches in this high‐risk disease, may be considered in future prospective studies to improve outcomes.

### 4.7 Implications for Molecular Risk Stratification

Current systems including DIPSS‐plus and MIPSS70+v2.0 do not account for *TP53* allelic status or VAF as independent variables. This builds a compelling case for a *TP53*‐specific risk stratification in MPN. Specifically, multi‐hit *TP53* should be considered a higher risk molecular feature in CP‐MPN requiring enrollment in clinical trials of novel *TP53*‐directed agents.

### 4.8 Limitations

#### 4.8.1 Several Limitations Merit Acknowledgment

First, all included studies were retrospective and observational, introducing selection bias toward advanced‐phase patients more likely to undergo comprehensive molecular profiling. Second, marked heterogeneity across studies produced uniformly high *I* ^2^ values that limit the precision of pooled estimates. Third, only one study reported leukemic transformation rates in the *TP53*‐mutated subset, limiting conclusions on this critical endpoint.

Formal statistical assessment of publication bias was not performed given the small number of included studies and the descriptive nature of the pooled analysis. Conference abstracts were not excluded from the search strategy, to minimize the risk of missing unpublished or early‐reported data. Nonetheless, publication bias toward reporting *TP53*‐mutated cases in advanced or high‐risk clinical settings remains a concern and is acknowledged as a potential source of overestimation of mutation frequency and adverse outcome rates in this analysis.

## 5 Conclusion

This systematic review demonstrates that somatic *TP53*‐mutations are a rare but clinically significant molecular finding in Ph–negative MPN, with strong enrichment in advanced disease phases—particularly MF and AP/BP‐MPN. The frequent multi‐hit allelic configurations, high mean VAF of 37.48%, and frequent association with unfavorable cytogenetics collectively mark a state of profound genomic instability with high leukemic transformation potential. Survival outcomes are strikingly poor in AP/BP‐MPN and substantially shortened in CP‐MPN, contrasting with the preserved OS observed in *TP53*‐mutated PV and ET.

The consistently low allo‐HCT utilization rate despite aggressive disease biology highlights a critical unmet need. Earlier molecular identification of *TP53*‐mutations, systematic allelic status and cytogenetic risk, and timely transplant referral before AP/BP‐MPN evolution are imperative. Integration of *TP53* mutational status—particularly allelic configuration and VAF—into prospective MPN risk stratification models should be a priority for the field.

Future work must address the limitations of the current evidence base through prospective multi‐center registries with standardized NGS panels, uniform definitions of multi‐hit *TP53*, serial VAF monitoring, and pre‐specified survival and transformation endpoints stratified by allelic status and disease phase.

## Author Contributions

Literature search, data extraction, manuscript drafting, and critical review: **Mahmood Aldapt**. Data analysis, manuscript drafting, and critical review: **Rasha Kaddoura**. Data screening, study selection, and data collection: **Ahmed O. Saleh**, **Mostafa Najim**, and **Ahmad Salem**. Data collection and quality assessment: **Abdulrahman F. Al‐Mashdali**. Quality assessment and publication bias assessment: **Bassam Muthanna**. Data collection: **Ruba Adel Aweer** and **Marrita Rabadi**. Manuscript writing, critical review, and overall supervision: **Shehab F. Mohamed**. All authors read and approved the final manuscript.

## Funding

This research is funded by The Pennsylvania State University.

## Ethics Statement

The authors have nothing to report.

## Consent

The authors have nothing to report.

## Conflicts of Interest

The authors declare no conflicts of interest.

## Supporting information




**Supporting File**: jha270385‐sup‐0001‐SuppMat.docx.

## Data Availability

All data supporting the conclusions of this study are extracted from previously published studies included in this systematic review. No new primary data were generated. The data extraction framework is available from the corresponding author upon reasonable request.
