# Supplementary material for: Clinicopathologic Characteristics of Somatic TP53 Mutations in Philadelphia Chromosome–Negative Myeloproliferative Neoplasms: A Systematic Review
Source: EJHaem. 2026 Sep 19;7(5):e70385. doi: 10.1002/jha2.70385 (PMC13589805; doi:10.1002/jha2.70385)
Supplement: Supplementary file 1 [file JHA2-7-e70385-s001.docx]

Supplementary material

**Demographics**

Figure S1. Pooled age variable

Figure S2. Pooled male variable

Figure S3. Pooled female variable

**TP53 mutation prevalence**

Figure S4. Pooled TP53 mutation prevalence

Figure S5. Pooled TP53 mutation in MF or AP/BP

**Disease phase distribution**

Figure S6. Pooled MPN in AP/BP variable

Figure S7. Pooled Primary MF variable

Figure S8. Pooled ET variable

Figure S9. Pooled PV variable

**DIPSS+ risk stratification**

Figure S10. Pooled MF DIPSS+ high variable

Figure S11. Pooled MF DIPSS+ int2 variable

Figure S12. Pooled MF DIPSS+ int1 variable

**TP53 allelic status**

Figure S13. Pooled TP53 multi-hit

Figure S14. Pooled TP53 single hit

**VAT**

Figure S15. Pooled TP53 VAT

**Cytogenetic Risk**

Figure S16. Pooled very high cytogenetic risk

Figure S17. Pooled unfavorable cytogenetic risk

Figure S18. Pooled favorable cytogenetic risk

**Allo-HCT utilization**

Figure S19. Pooled bone marrow transplant

Figure S20. Leukemic transformation

**Table S1.** Newcastle-Ottawa Scale Quality Assessment of Included Studies

| **Study** | **SELECTION** | | | | **COMPARABILITY** | | **OUTCOME** | | | **Total Score** | **Risk Category** |
| --- | --- | --- | --- | --- | --- | --- | --- | --- | --- | --- | --- |
|  | **Representativeness of Exposed Cohort** | **Ascertainment of Exposure** | **Selection of Non-Exposed Cohort** | **Outcome Not Present at Start** | **Comparability (Age)** | **Comparability (Other Factors)** | **Assessment of Outcome** | **Follow-Up Duration** | **Follow-Up Adequacy** |  |  |
| **Fathima, 2025** | **✓** | **✓** | **✗** | **✓** | **✓** | **✓** | **✓** | **✓** | **✗** | **7** | **Low** |
| **Gagelmann, 2023** | **✓** | **✓** | **✓** | **✓** | **✓** | **✓** | **✓** | **✓** | **✗** | **8** | **Low** |
| **Grinfeld, 2018** | **✓** | **✓** | **✓** | **✓** | **✓** | **✓** | **✓** | **✓** | **✗** | **8** | **Low** |
| **Kim, 2022** | **✗** | **✓** | **✓** | **✓** | **✓** | **✓** | **✓** | **✓** | **✗** | **7** | **Low** |
| **McNamara, 2018** | **✓** | **✓** | **✗** | **✓** | **✓** | **✓** | **✓** | **✓** | **✗** | **7** | **Low** |
| **Tefferi, 2025** | **✓** | **✓** | **✓** | **✓** | **✓** | **✓** | **✓** | **✓** | **✗** | **8** | **Low** |
| **Rolles, 2026** | **✓** | **✓** | **✓** | **✓** | **✓** | **✓** | **✓** | **✓** | **✗** | **8** | **Low** |
| ***Badar, 2025*** | *Not assessed — Letter to the Editor* | | | | | | | | | | |
| ***Marcellino, 2023*** | *Not assessed — Letter to the Editor* | | | | | | | | | | |
| ***Roncoroni, 2022*** | *Not assessed — Conference Abstract* | | | | | | | | | | |
| ***Son, 2023*** | *Not assessed — Conference Abstract* | | | | | | | | | | |
